# The Zebrafish as a Model for Ocular Translational Research: From Retinal Repair to Regeneration

**DOI:** 10.3390/cells14171405

**Published:** 2025-09-08

**Authors:** Bijorn Omar Balzamino, Mariagrazia Severino, Concetta Cafiero, Marco Coassin, Antonio Di Zazzo, Alessandra Micera

**Affiliations:** 1 Research and Development Laboratory for Biochemical, Molecular and Cellular Applications in Ophthalmological Science, IRCCS-Fondazione Bietti, 00184 Rome, Italy; bijorn.balzamino@fondazionebietti.it (B.O.B.); mariagraziaseverino@gmail.com (M.S.); 2 Medical Oncology, SG Moscati Hospital, 74010 Statte, Italy; concettacafiero@gmail.com; 3 Ophthalmology Operative Complex Unit, University Campus Bio-Medico, 00128 Rome, Italy; m.coassin@policlinicocampus.it (M.C.); a.dizazzo@policlinicocampus.it (A.D.Z.)

**Keywords:** zebrafish, retina, regeneration, retinogenesis, gliosis, Müller cells, animal model, precision medicine

## Abstract

In the last years, the zebrafish model has become a primary model system for vertebrate tissue regeneration, particularly for neurodegeneration and metabolic disease. Zebrafish (*Danio rerio*) are small freshwater teleosts valued for disease modelling, which are widely used in genetic laboratories, as a key model for studying neurodegenerative, metabolic, cardiac and dystrophic diseases, supporting the goal of identifying new therapeutic targets and approaches. Zebrafish can proliferate and produce/regenerate neurons. In response to retinal injury, zebrafish can regenerate multiple classes of retinal neurons and particularly, Müller glia-derived progenitor cells (MGPCs) can regenerate all types of neurons and restore visual function upon injury. The Jak/Stat-pathway of zebrafish retina represents one of the cell-signalling pathways involved in reprogramming Müller glia into MGPCs. In this era characterized by a revolution in experimental models and the future of omics, zebrafish might represent a suitable animal model for studying retinal degeneration and regeneration. In this context, the review is not meant to be entirely comprehensive of the zebrafish field, but it will highlight the usefulness of this model in discovering some mechanisms underlying retinal repair and regeneration.

## 1. Introduction

Teleosts, and particularly zebrafish, excel as model systems for the study of tissue development, including retinogenesis [1,2]. Optically transparent fish develop externally and quickly, facilitating experimental manipulations and observations [3]. A comprehensive and high-quality genome sequence is available for zebrafish, showing significant similarity to the human one, and excellent technologies have been developed to create stable transgenics, allowing for the identification and characterization of genes that are important for development [1,4]. As zebrafish continue to grow, the teleost retina experiences persistent neurogenesis, which makes it an excellent research tool to understand the process of neurogenesis in postembryonic mammals, as well as how retinal regeneration occurs in response to acute damage [2,5].

The zebrafish retina is an excellent model for research on vision depletion. Rod and cone photoreceptors on zebrafish retina are maximally sensitive to long wavelengths (red), middle wavelengths (green), short wavelengths (blue), and ultraviolet wavelengths (UV) [6,7]. Genetic approaches, NGS approaches, and the comprehension of physio-pathological mechanisms can be useful to better understand the main age-related retinal and macular disorders.

In view of this, this review aims to explore the current state and future potentiality of zebrafish as a versatile in vivo model for studying retinal disorders and testing novel therapeutic strategies. By intersecting tissue engineering, drug delivery, and neuro-regenerative biology, we highlight how zebrafish models can clarify retinal development and degeneration and can contribute to our understanding of repair and regeneration. Particular attention was devoted to the roles of Müller glia, microglia, and epigenetic regulation, as well as the application of omics technologies to uncover novel cellular pathways and therapeutic targets. This synthesis intends to offer a comprehensive perspective on the translational value of zebrafish in vision science and regenerative medicine.

An extensive literature review of papers published in a range between 2000 and 2025 was performed based on a standard procedure (PubMed (https://pubmed.ncbi.nlm.nih.gov/; accessed on 24 April 2025), Embase (https://www.embase.com/; accessed on 8 April 2025), and Google Scholar (https://scholar.google.com/; accessed on 16 April 2025)) to select the manuscripts of interest. PubMed search terms included “zebrafish”, “retina”, “retinopathy”, “ocular models”, “retinogenesis”, “repair”, “neurodegeneration”, “Müller Cells”, “biological markers”, and “personalized medicine”, either alone or in combination with the search term “Zebrafish” (last accessed on 25 July 2025). The review will introduce zebrafish as a useful and smart experimental model for understanding the mechanisms underlying physio-pathological states and learning the repair/regenerative mechanisms to be translated into clinics in the near future (individualized eye medicine).

## 2. Age-Related Eye Diseases: Therapeutic Routes and Experimental Models

Due to the increase in life-span and the effects of light, pollution, and changes in lifestyle, as well as work-related factors, age-related eye diseases are becoming more prevalent, such as myopia, glaucoma, age-related macular degeneration (AMD), epiretinal membranes, and cataracts [8,9,10,11,12]. Current treatments range from tissue substitution with polymers to topical eye drops and intravitreal injections [13,14]. A summary of the main contributions of the zebrafish model regarding the major retinal diseases is reported in Table 1.

### 2.1. Therapeutic Approaches

The use of topical treatments is often associated with the erosion of the surface epithelium and patients refer ocular surface discomfort to dry eye syndrome, as reported in the recently defined urban eye syndrome [20]. In addition, the increasing surgical treatments related to intravitreal injections to control angiogenic levels are debilitating and often do not cure the disease. To overcome treatment challenges, research and development efforts have increased by using polymers that allow a prolonged drug release, implementing drug delivery as well as improving the patient use and treatment compliance. Recent studies demonstrate the application of biopolymers and biomaterials in various forms [21,22]. Zebrafish also find applications in retinal and optic nerve degeneration and potential regeneration [23]. Corneal transparency, corneal oedema, and corneal melting represent a challenge, and the high immunological risks might provide some difficulties [24].

Official therapeutics for the treatment of ocular diseases include steroids, antibiotics, and biological pharmaceuticals (neutralizing anti-VEGFs antibodies) [21]. Experimental therapeutics presently investigated include antioxidants, complement factor inhibitors, and innovative therapeutic approaches (stem cells, and viral vectors for gene therapy) [25,26].

Local delivery is often crucial as it includes topical eye administration (eye drops), subconjunctival injections, or intravitreal injections that provide short-term aid and require numerous administrations [27]. Recent treatments use polymers to extend delivery duration in the eye, and widespread research is being conducted to reduce application frequency. Recent improvements have focused on new challenges through both in vitro and in vivo studies [28]. This review covers recent work aimed at evaluating the potential therapeutic contribution of the zebrafish model in eye disorders. Furthermore, we discuss encouraging approaches in tissue engineering techniques in this in vivo animal model. An alternative route to deliver drugs to the eye is by the injection of pharmacologic agents. By subconjunctival injection, drugs can be released next to the sclera without being blocked by the cornea [29]. Due to the permeability of the scleral layer, drugs can be easily delivered to the interior of the eye, particularly the posterior segment [30,31]. The subconjunctival drug injection or implant procedure is relatively more invasive than the use of eyedrops but guarantees a prolonged drug delivery in comparison to eyedrops, lasting up to several months between injections [27]. It would be significantly easier to maintain daily eyedrop administration regimens with minimally invasive injections or implantations every few months [27]. There are still challenges associated with this method; however, since the subconjunctival space is not as drained as the anterior surface, it is still replete with drainage pathways. Blood vessels in the conjunctiva and sclera, as well as lymphatic drainage, will interfere with dose delivery, causing the drug to enter the bloodstream rather than penetrating the sclera and entering the eye [30].

### 2.2. Experimental Models in the XXI Century: Monolayers, Organoids, and the 3Rs Refined In Vivo Experiments

Up to the present time, monolayers, organoids, and animal models have offered plain advantages and disadvantages as research tools for understanding cell-to-cell and cell-to-microenvironment interactions, particularly for disease modelling, understanding cell-to-mediator interactions, identifying biomarkers, and informing drug discovery. In animal research, the “3Rs”, referring to replacement, reduction, and refinement, have gained preponderant importance and are devoted to minimizing the use of animal research models while maximizing the scientific value of statistically significant results [32]. Monolayers are cheaper and easy to use, but the absence of the complexity and interactions of the 3D structure of real tissues limits their ability to mimic in vivo conditions. Organoids resemble both the structure and function of organs, allowing for more accurate disease modelling and drug testing, but greater challenges are encountered and more expertise and expensive tools are required, and sometimes they cannot replicate the complexity of an in vivo system (cell-to-cell and cell–mediator interactions) [33]. Retinal organoids can mature over several months in culture, with the photoreceptors expressing specific markers like recoverin and rhodopsin [33]. Retinal organoids are 3D cell cultures derived from stem cells that mimic the structure and function of the human retina. These retinas in miniature contain various retinal cell types, such as photoreceptors, bipolar cells, horizontal cells, amacrine cells, ganglion cells, and Müller cells, arranged in a layered structure similar to the native retina [34]. This organized culture is a valuable tool for studying retinal development, disease modelling, and drug discovery since the cell-to-cell and cell-to-mediator interplays are like those of native tissues [34]. Data integration from the single-cell profiling of human organoids and of human primary tissue samples can provide complementary information, allowing each approach to play to its strengths. Both monolayers and organoids can be used to generate cultured cells by digesting and seeding cells derived from biopsies to allow personalized medicine approaches or studies of disease-specific responses. Organoids and monolayers can be used in concert, providing a simple and controlled environment for studying specific aspects of the pathological microenvironment. Of interest, they can be derived from patient-specific cells, offering potential for personalized medicine or for developing scaffolds for reconstructive medicine, such as penetrating keratoplasty (PK) [33]. The cornea is a window of transparent tissue at the front of the eyeball, which allows light to pass into the eye and provides focus so that images can be seen. Various diseases or injury can make the cornea either cloudy or out of shape, reducing the physiological passage of light, and affects vision [35]. Finally, animal models provide the most comprehensive representation of human physiopathology, allowing for studies of complex interactions between different organs and systems, but these models need animal facilities and expert users and might be expensive. Moreover, the use of experimental models raises ethical concerns and sometimes they may not always accurately predict human responses due to differences in physiology and genetics.

Zebrafish models have some advantages over rodent and murine models as summarised in Table 2, such as longer survival and the availability of transgenic lines marking specific bone cell types combined with the transparency of early-life stages allowing for the in vivo observation of the dynamics of skeletal development [36].

Glaucoma and diabetic retinopathy are neurodegenerative retinal diseases that gradually destroy retinal neurons. A neuroprotective or regenerative strategy for neuronal loss is often sought after because central nervous system neurons cannot regenerate in mammals. In neuroprotection, neurons that have survived harmful insults are preserved in structure and function, whereas in regenerative approaches, neurons are replaced or rewired that have been lost, or damaged axons and dendrites are grown again [17]. In vitro microenvironments are obtained under standardized laboratory settings designed to mimic the natural cellular environment, crucial for studying cell behaviour and interactions [37]. Mimicking cellular environments is necessary for understanding how cells respond to different conditions and how these conditions affect their function [37]. While some microenvironments are relatively simple, others such as that of the retina are complex and enriched with different cell subtypes interacting and providing functional networking [38]. Müller glia, as major glial cell types in the retina, maintain retinal homeostasis and provide structural support to retinal photoreceptors and retinal ganglion cells [39]. This tidy, complex retinal tissue can be offended by different insults (physical, chemical, or biological) and can repair itself due to its regenerative potential [40]. In zebrafish, reactive Müller cells are characterized by reprogramming reactions that result in the proliferation of neural progenitors that can regenerate retinal cells [41,42]. Müller glia reprogramming and regeneration in mammals has been uncovered by several recent studies in fish, which might shed light on their regenerative capacity [41,42]. Mammalian Müller cells can adopt stem cell characteristics and can be induced to proliferate and regenerate under special conditions, although their regeneration capacities are limited by the microenvironment [43,44]. Zebrafish have shown an incredible ability to regenerate their retina after injury, so self-renewing retinas are not unreachable [17]. In the retina, Müller cells and astrocyte-like radial cells play a chief role in maintaining retinal structure and homeostasis [45]. Upon retinal injury, Müller cells acquire a stem cell phenotype by undergoing reprogramming events leading to retinal regeneration, due to progenitor cell proliferation [42,46]. Unfortunately, this regeneration potential is absent in mammalian systems for unknown reasons, despite being present in all vertebrates [43]. We might be able to understand why mammalian cells lack the regeneration ability of fish and how we may encourage it by understanding the mechanisms behind this fish regenerating potential.

## 3. Properties of Zebrafish Model: Retina Development and Degeneration Interplays

The zebrafish retina is composed of various cell types, including photoreceptors, retinal ganglion cells, amacrine cells, and Müller glia, mirroring the structure of mammalian retinas. Unlike mammals, zebrafish retinas can regenerate damaged tissue, with Müller glia playing the pivotal role by reprogramming into retinal progenitor cells and generating new neurons [40]. Human retinas contain more rods than cones, like murine ones (~95% rods and ~5% cones), and the fovea (the central area of the retina that is responsible for visual acuity) is primarily populated by cones [47]. On the contrary, zebrafish have a cone-dominated retina (around 65% of rods and 35% of cones), like the central human retina [48]. In addition, the zebrafish retina displays regional localization and molecular specializations of cones that are like those of primate fovea [49], providing a useful model to understand the molecular mechanisms involved in the development and regeneration of the central retina. Retinal degenerative models of zebrafish provide easy and quick research studies with opportunities not only to characterize retinal diseases but also to identify mechanisms of photoreceptor regeneration. Research on zebrafish degeneration and regeneration could lead to the development of new treatments for counteracting the retinal degeneration in the main retinal and vitreoretinal disorders, such as in the case of age-related macular degeneration, retinitis pigmentosa, and other blinding conditions [15]. Table 3 provides a comparison between zebrafish and other animal model applications in ophthalmic research.

Multiomics and next-generation sequencing (NGS) technologies have greatly enriched our understanding of genetically based retinal diseases and widened the perspectives of study, starting from simple factors to complex interacting molecules. Biostatistics and mathematical modelling can benefit from these methods, but a gap exists between the amount of data generated and the ability to evaluate the predictions made. The importance of this breakthrough technology lies in the fact that it provides an ever-expanding number of model species with the potential to be treated by reverse genetics.

Zebrafish hmx1 promotes retinogenesis, while the Fgf signalling plays crucial actions in morphogenesis [51]. During embryonic lens and retina development, Fgf19 was involved in cell survival but not cell proliferation, and was mainly expressed by the nasal retina and lens [52]. In the retina, Fgf19 was found in differentiating lens fibre cells but not in neuronal differentiation and lamination [52]. The loss of nasal fate in the retina caused by Fgf19 knockdown, the expansion of nasal fate in the retina caused by Fgf19 overexpression, and eye transplantation indicated that Fgf19 is crucial for the nasal-temporal patterning of the retina that is critical for the guidance of retinal ganglion cell axons [52]. Fgf19 knockdown also caused incorrect axon pathfinding, further sustaining that Fgf19 positively regulates the patterning and growth of the retina, as well as the differentiation and growth of the lens in zebrafish [52].

Previous authors have unbiasedly studied zebrafish retinal development and function through genetics [53]. Fewer types of genetic retinal disease models are available in zebrafish than in mammalian models. Due to multiplexing, mutations can be introduced at multiple target sites simultaneously, observed rapidly, and analysed for studying retinal regeneration, but they are insufficient for mimicking chronic diseases with degeneration kinetics like those of humans [2,54]. Some studies have shown that zebrafish can regenerate their retina after exposure to aluminium, which causes retinal neurodegeneration [55].

Multiple-omics approaches generate and analyse massive amounts of epigenetic and gene expression data about the retina [56,57]. Since so much starting material is required for these experiments, they still heavily rely on whole eyes or retinal tissues, or, in some cases, genetically or transgenically purified cell types. While bulk sequencing data is informative, they tend to overlook much of the genetic and epigenetic information within each individual cell; the data often contain averaged signals from heterogeneous populations and, at times, different developmental ages of cells [58]. Molecular tools, such as genomics and epigenomics, can be combined with cell-specific transgenic reporter lines, making zebrafish an ideal model for those types of experiments in the future. The purpose of this approach is to identify new types of cells, intermediate types of cells, or new genes and regulatory pathways that are not detectable by bulk profiling.

The zebrafish retina quickly develops to reach an adult-like structure within 72 h post-fertilization. Zebrafish larvae are used to study the high metabolic demands of photoreceptors and the interplay between photoreceptors and the retinal pigment epithelium (RPE) during retinogenesis and in mature retinas [59]. The transcription factor Egr1 plays a role in early zebrafish retinogenesis, particularly in the differentiation of specific retinal cell types and their connections [60]. Studies have explored the role of cadherin-6, a cell adhesion molecule, in the development of specific retinal cell types in zebrafish [61]. Zebrafish are robust genetic tools and attractive models for neuroprotection studies because of their wide range of functional and behavioural tests and their low cost [62]. Zebrafish are often exposed to intense light, chemical pollution, and mechanical damage as insults to model retinal disease and study neuroprotection [17]. Experimental models of retinal neuronal damage are essential for assessing the neuroprotective, reparative, or regenerative potential of a given agent or candidate gene. Identifying drugs or genetic targets for retinal regeneration or protection requires low-cost and high-throughput screening techniques [33]. This type of screening analysis is ideal for zebrafish, which are compact, low-cost, and have robust genetic tools [63]. In addition, zebrafish share the same eye structure and development as humans and have cone-dominated retinas [64]. Zebrafish have thus become one of the most important tools for studying retinal neurodegenerative diseases [64].

Regarding adult neurogenesis, zebrafish allow for the investigation of adult NSPC heterogeneity and cell cycle dynamics at a level of detail that is difficult to achieve in mammals, thanks to adult neurogenic niches easily accessible to live imaging [65]. Upon behaviours and response to various stimuli, adult NSPCs can be recapitulated in zebrafish, and the regenerative capacity of zebrafish neurogenesis may help to understand the lack of regeneration in the mammalian brain [40,65]. In zebrafish, the prompt response to injury is similar regardless of injury mode and occurs with reactive glia that become proliferative and enter a neurogenic state [66]. Once reactive Müller glia become multipotent progenitors in zebrafish, they give rise to all retinal neurons irrespective of injury, proliferating more in insulted areas (populated by death cells) with respect to those of intact retina [41]. The greatest difference between regenerating and non-regenerating species seems to lie in the capacity of Müller glia to switch into proliferating progenitors, with less emphasis on the injury mode. In both zebrafish and mice, the transcriptomic profiles of resting and reactive Müller glia are similar following injury [66].

The cellular niche, the site of retinal progenitor cells in growing and regenerating adult zebrafish retina, was examined for its molecular profile upon the stimulation of injury-induced proliferating progenitor cells, a process that gives rise to regenerated cone photoreceptors, and a simple heat lesion paradigm was introduced for analysing retinal regeneration [55]. In differentiated retina, the microenvironment created by the peripheral CMZ, some Müller cells contribute to the survival of multipotent, self-renewing stem cells [67].

Key roles in retinal neurogenesis and in retaining synaptic integrity have also been attributed to microglia, the intrinsic macrophages of neuronal tissues, located at the interface of the nuclear and synaptic layers [68]. In the zebrafish retina, microglia undergo rapid and significant changes in morphology, shifting from ramified to ameboid, to migrate to sites of cell death [69]. At the same time, microglia display a fast induction of genes encoding for inflammatory cytokines [69]. In zebrafish, the essential role of microglia during retinal regeneration has been demonstrated by studies using genetic and pharmacological techniques to ablate it. The depletion of microglia leads to a reduction in Müller glia proliferation [70]. In zebrafish, when the Colony Stimulating Factor-1 receptors (CSF1R) are knocked down, the retinal progenitor cells maintain continuous proliferation (without microglia receptor), leading to a delayed neurogenesis [71]. This impairment would suggest that microglia contribute to neurogenesis by signalling neural progenitors to exit the cell cycle and the reoccupation of microglia partially restored neurogenesis [71].

**Figure 1:**
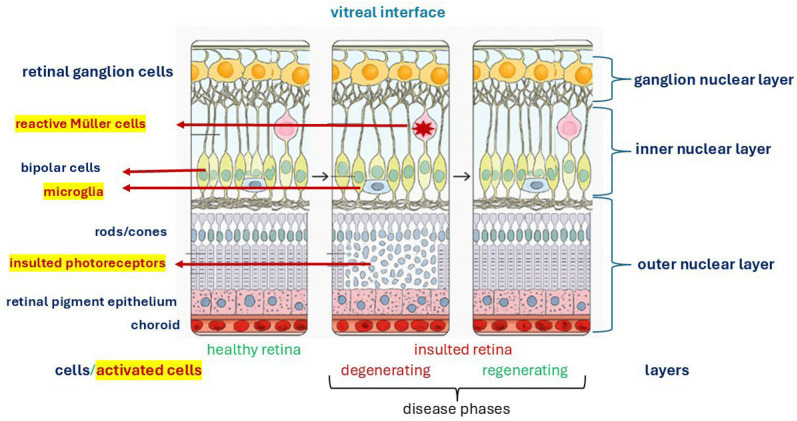
Retinal insult and remodelling: a generic insult can develop in two major phases involving cell activation and tissue-matrix regeneration retaining the physiological function. This image was created with BioRender (https://biorender.com/, accessed on 1 September 2025).

Although ideal and described in animal models, the major steps occurring at the insulted retinal level and the cells involved in these microenvironmental changes and tissue-function recovery are schematized in Figure 1.

## 4. Genetic and Epigenetic Factors Regulating Retinal Neurogenesis: From a Zebrafish Point of View

**Figure 2:**
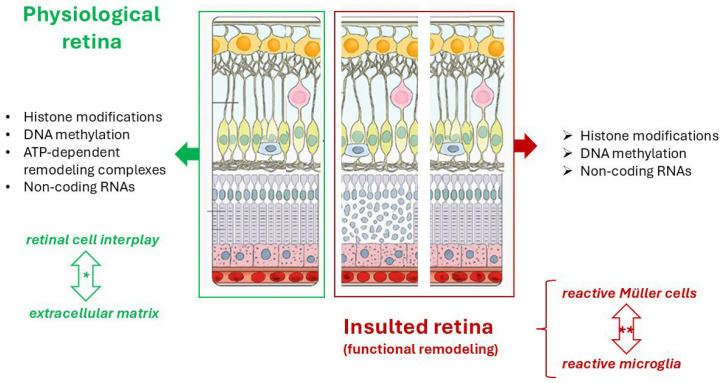
Schematic representation of the epigenetic factors participating in physiological homeostasis cell-to-cell and cell-to-matrix interplay and retinal regeneration upon insult in adult zebrafish. DNA methylation, Histone modifications and ATP-dependent remodeling complexes are mechanisms of chromatin remodeling. The asterisk (*) indicates a homeostatic crosstalk between retinal cells and extracellular matrix while (**) point at the crosstalk between reactive Müller cells and activated Microglia in the attempt to restore a physiological retina upon insult, by providing neuroprotection and promoting repair to restore physiological conditions. This image was created with BioRender (https://biorender.com/, accessed on 4 September 2025).

Zebrafish models have become popular for studying cancer and cardiovascular disease genetics and epigenetics [72]. Zebrafish are a valuable model organism for studying human diseases, as they share a high degree of genetic similarity with humans. More than 70% of human protein-coding genes and 82% of human disease-related genes have zebrafish orthologs, including most of the genes involved in skeletogenesis and retinogenesis [73]. DNA, RNA, and SNPs as well as chromatin abnormalities can provide interesting insight into the mechanisms of tumorigenesis and there are many techniques for analysing tumour genomes in zebrafish, including array-based methods as well as next-generation sequencing technologies. Genome-editing with CRISPR/Cas9 is a fast and easy method to create knockout alleles and stable zebrafish mutant lines [16], as depicted in Figure 2.

Genetics, development, and readily available ophthalmic tools make zebrafish ideal for investigating retinitis pigmentosa (RP) mechanisms and potential treatments. RP causes rod photoreceptor death and progressive vision loss, leading to blindness, due to a genetically based retinal degeneration process [74]. Zebrafish and human retinas share a significant number of genes, including those implicated in RP [75]. These genetic similarities point at the zebrafish models with mutations in genes known to cause RP in humans. Since zebrafish develop externally, it makes it easier to observe and study their retinas during development and adulthood. Zebrafish have a cone-rich retina, like humans, which is important for studying cone-related aspects of RP [75]. To better highlight the current usefulness of the zebrafish model in basic and translational human research, the main gene manipulations, to mimic human retinal disease, are listed, emphasizing the zebrafish contribution (Table 4).

### 4.1. Genetic and Epigenetic Factors

A considerable proportion of phenotypic variability during evolution is caused by coding and non-coding DNA mutations [77]. An ocular malformation can have different causes, and its phenotypic expression may vary greatly [78]. This process is influenced by epigenetic factors [79]. Inheritance occurs through a recessive, dominant, and X-linked mechanism, but in many cases the inheritance pattern is sporadic and not Mendelian [80]. Several environmental factors can contribute to this condition, such as viral infections, irradiation, and drug consumption during pregnancy [81].

A majority of microphthalmias and anophthalmias have been linked to mutations in transcription factors. Recently, the transforming growth factor (TGF) superfamily has been linked to microphthalmia/anophthalmia, playing a critical role in embryonic development, regulating cell proliferation, and apoptosis, as well as in defining the neural crest, forming bone, and assisting in organogenesis; these cells play a role in the development of embryos [50].

A zebrafish model makes it much easier to examine how small molecules may influence biological processes in vivo through chemical genetic screens [82]. Zebrafish embryos provided new insights into vertebrate development by chemical genetic screens [83]. As in mammals, only the forebrain possesses dopaminergic neurons (DAs), and these are found in the olfactory bulb, retina, preoptic area, pretectum, ventral diencephalon, and hypothalamus. Noradrenergic neurons (NAs) and dopaminergic neurons (DAs) contain neuromodulators derived from tyrosine, which together form catecholaminergic neurons (CAs). Although zebrafish and mammals evolved at a great deal of evolutionary distance, they share anatomical, molecular, and functional homologies across the forebrain DA groups of zebrafish and mammals [83,84].

Even though we have a good understanding of how the retina develops and the intrinsic and extrinsic factors involved in this development, we know truly little about how these factors are regulated and how they are activated. The epigenetic mechanisms contributing to retinal development have been gaining increasing attention in recent years, which further complicates the process.

### 4.2. Histone Modifications and Changes in the Chromatin Landscape

In zebrafish, histone deacetylase 1 (HDAC1) activity is pivotal for promoting neural progenitors by antagonizing the gene expression of Notch targets, by modulating the expression of canonical and non-canonical Wnt genes [85]. In the hindbrain, HDAC1 mutations cause a significant reduction in cell proliferation [86]. In contrast, histone HDAC1 regulates Notch and Wnt pathway activity during retinal development to promote cell cycle exit and regulate retinal neurogenesis [87], thus highlighting the importance of histone modifications during retinal development. Mutant retinal cells that are missing HDAC1 fail to differentiate [88].

A possible epigenetic modification would further facilitate the ability of MGs to immediately reprogram and regenerate damaged cells after injury, consistent with their multipotency [89].

### 4.3. DNA Methylation and Hydroxymethylation

In mammalian development, methylation is crucial for the epigenetic regulation of gene expression, and this must be maintained during cell division as well. There is a limited understanding of how DNA methylation and hydromethylation work during retinal development. In zebrafish, DNA methylation and hydromethylation-related genes are expressed in proliferating retinal and lens cells during development [90]. Accordingly, DNA methylation plays a role in teleost eye development, and DNA methylation is responsible for eye diseases. Adaptive evolution can be accelerated by small genetic changes that alter epigenetic regulation because they trigger dramatic changes in the expression of large sets of genes, despite the well-documented role that DNA methylation plays in development and disease. The positioning of nucleosomes, the compaction of chromatin, and the access of transcription factors to DNA are affected by histone modifications and DNA methylation. In modern science, epigenetic marks work in tandem with genetic codes to determine the activity of genes, whether they are active, silenced, or inactive, but poised for activation when appropriate conditions arise. The chromatin environment can be altered through modifications to histones [91] and changes in the DNA methylation and hydroxymethylation landscape [92].

## 5. Retinal Degeneration, Regeneration, and Engineered Approaches: The Contribution of Zebrafish

The goal of retinal tissue engineering is to provide patients with retinal disorders with alternatives to regenerating their damaged retinas in whole or in part. Zebrafish are widely used as retinal disease modelling systems [93]. The zebrafish retina is comparable to other vertebrates [40]. Zebrafish retinas are similar to human retinas and more than 70% of human genes have at least one ortholog in zebrafish [94]. Human retinitis pigmentosa-associated genes listed in RetNet (https://sph.uth.edu/retnet/, accessed on 2 September 2025) are preserved zebrafish orthologs. The retina develops completely in 5 days [95], has a small size, and is simple to quantify [95].

**Figure 3:**
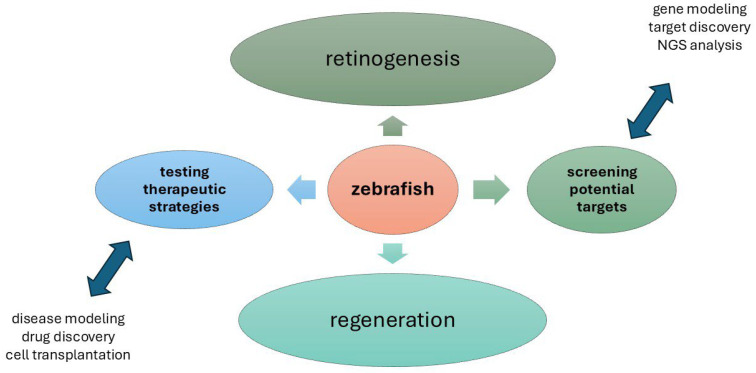
Overview of present and potential zebrafish applications in basic, applied, and translational Zebrafish eye research. Retinogenesis and regeneration upon physiological and pathological states represent the major fields of investigation in zebrafish model. The main actions are testing therapeutic strategies and screening the potential targets represent the goals that hopefully can be achieved in a shorter time by using this animal model. NGS, Next-Generation Sequencing. (Microsoft PowerPoint, Office 365; Redmond, WA, USA).

The complex cell behaviours and genetic regulation, as depicted in Figure 3, required for retinal morphogenesis, are still poorly understood [96]. Zebrafish eye development is regulated by several familiar genetic screens [53]. To identify factors that regulate eye morphogenesis, a forward genetic, small-scale haploid mutagenesis screen was performed in zebrafish [97]. In total, about 100 genomes were screened as well as isolated shutdowns [97]. During eye morphogenesis, cell proliferation, cell death, and tissue defects have been analysed using live imaging and antibody staining. CRISPR-Cas9 has been used to engineer the largest deletion mutants in zebrafish, which are less than 300 kb in size [98].

Biological functions such as cellular differentiation, immunomodulation, and neovascularization are mediated by exosomes, which are a subset of extracellular vesicles containing biomolecules such as proteins, nucleic acids, and lipids [99]. To investigate the role of exosomes in ocular disorders [99], zebrafish exosomes are being studied, due to their involvement in diseases such as neurodegeneration, cardiovascular disease, and cancer [100]. There are various challenges in diagnosing and treating ocular diseases such as age-related macular degeneration, glaucoma, infectious endophthalmitis, diabetic retinopathy, autoimmune uveitis, etc., because it is difficult to obtain samples or because they seem to be masquerading syndromes as they are often unidentifiable [18].

## 6. Concluding Remarks and New Perspective

To date, there is a gap in the literature regarding retinal neuroprotection and regeneration. In recent years, zebrafish have emerged as an exceptionally informative model for investigating retinal development, degeneration, and regeneration. Genetic and structural similarities to humans, combined with remarkable regenerative capacities, allow zebrafish to provide important insights into the cellular and molecular processes that sustain vision. A unique aspect of zebrafish retinas is the capability of regenerating after injury or pathological insults, which makes the zebrafish a unique resource for identifying the mechanisms of regeneration that might contribute to the development of neuroprotection and the restoration of vision. The use of zebrafish has revealed that Müller cells possess an untouched regenerative ability that, if better understood, could provide targets for cell-based therapies for retinopathies. Moreover, the growing integration of omics approaches (transcriptomics, epigenomics, proteomics, lipidomics, and metallomics) is opening up new opportunities for dissecting complex regulatory networks involved in retinal health and disease. In combination with zebrafish models, these methodologies can lead to a prediction and mechanistic understanding of renal function and repair that moves beyond observational biology.

Taken together, zebrafish-based discoveries will need to be translated into clinical applications in the near future. Transforming basic discoveries into therapeutic strategies will be made possible by advances in gene editing, high-throughput screening, and single-cell technologies. Combining the strengths of zebrafish with emerging biotechnologies could lead to regenerative therapies for currently untreatable retinal diseases in the future.

## Figures and Tables

**Table 1:** Zebrafish contributions to the study of specific retinal diseases.

Retinal Disease	Zebrafish Contribution	Significance	References
Retinitis Pigmentosa (RP)	Generation of transgenic zebrafish lines with mutations in rhodopsin, PDE6, and other phototransduction genes.	Enables investigation of photoreceptor degeneration and potential gene therapy approaches.	[15]
Leber Congenital Amaurosis (LCA)	CRISPR/Cas9-induced mutations in genes such as CEP290 and AIPL1.	Provides insights into early-onset retinal dystrophy and validates targets for gene replacement strategies.	[16]
Age-Related Macular Degeneration (AMD)	Oxidative stress and light-damage models used to study retinal pigment epithelium (RPE) dysfunction.	Facilitates understanding of molecular pathways in RPE degeneration and screening of antioxidant compounds.	[10]
Diabetic Retinopathy (DR)	Hyperglycaemia-induced zebrafish models mimic vascular changes and neuronal dysfunction.	Allows study of early vascular pathology and high-throughput drug screening.	[17]
Glaucoma	Genetic models affecting intraocular pressure regulation (e.g., mutations in myocilin).	Provides a platform for studying optic nerve degeneration and neuroprotective strategies.	[9]
Cone–Rod Dystrophy	Mutant lines targeting cone photoreceptor genes (e.g., CNGB3).	Useful for exploring cone photoreceptor survival and regeneration potential.	[18]
Usher Syndrome	Knockdown and knockout models of usherin and harmonin genes.	Helps dissect mechanisms of photoreceptor–synaptic dysfunction and sensory cilia abnormalities.	[19]

**Table 2:** Summary of advantages, disadvantages, and limits of the three experimental approaches.

Model	Advantages	Disadvantages/Limits	References
In vitro monolayer	Monolayers are single layers of cells cultured on a flat surface, often used for studying cell behaviour, interactions, and drug permeability.	Simple to culture, easy to manipulate, and allows for high-throughput screening. May not accurately reflect the complexity of in vivo tissues, such as the three-dimensional structure and cell–cell interactions.	[33]
In vitro organoids	Organoids are 3D cell cultures derived from stem cells or progenitor cells that can self-organize into structures resembling organs.	More closely mimic the in vivo environment than monolayers, allowing for the study of tissue-specific functions and responses to stimuli. Can be more complex to establish and maintain than monolayers, and may still lack some of the complexities of in vivo tissues, such as the full range of cell types and interactions.	[34]
In vivo	In vivo models involve studying biological processes in living organisms, such as animals or humans.	Provide the most realistic representation of biological processes, allowing for the study of complex interactions between different tissues and organs. Can be more complex, costly, and ethically challenging to study, and may involve ethical considerations related to animal welfare.	[32]

**Table 3:** Comparison of zebrafish and other animal models in ophthalmic research.

Feature/Benefits	Zebrafish (*Danio rerio*)	Other Animal Models (Mouse, Rat, Rabbit, and Primate)	References
Genetic Manipulation	Highly open to genetic editing (CRISPR/Cas9, transgenesis); transparent embryos allow direct visualization of gene expression.	Genetic tools available (especially in mice), but less accessible in larger animals; visualization of early development is limited.	[16]
Developmental Transparency	Embryos and larvae are optically transparent, enabling non-invasive imaging of ocular structures.	Most mammalian embryos develop in utero, limiting in vivo visualization during early stages.	[50]
Ocular Anatomy	Retina shares conserved cell types and layered structure with humans. However, it lacks macula and fovea.	Mammals (e.g., primates) possess a macula/fovea, providing a closer anatomical resemblance to the human eye.	[40]
Regenerative Capacity	Robust retinal regeneration after injury, useful for studying repair mechanisms.	Limited or absent regenerative capacity in mammals, more representative of human pathology.	[40]
High-Throughput Screening	Small size, rapid reproduction, and low maintenance cost allow large-scale drug and genetic screens.	Larger animals are costly, time-consuming, and less practical for high-throughput studies.	[49]
Ethical Considerations	Generally considered less ethically constrained compared to higher vertebrates.	Ethical concerns are greater, especially for primates; strict regulations apply.	[32]
Translational Relevance	Excellent for mechanistic and developmental studies but some limitations in modelling human-specific ocular diseases.	Mammals, particularly primates, provide higher translational relevance due to closer ocular physiology and anatomy.	[48]
Cost and Maintenance	Low cost, minimal space, and straightforward husbandry.	Higher costs for housing, feeding, and care, especially in larger species.	[2,4]

**Table 4:** Genetically engineered zebrafish models and the related contribution to human retinal diseases.

Gene/Mutation	Human Retinal Disease	Zebrafish Contribution	References
rhodopsin (rho) mutations	Retinitis Pigmentosa (RP)	Models photoreceptor degeneration and progression of RP; used to test gene therapy strategies.	[74,75]
pde6c mutation	Cone Dystrophy/Achromatopsia	Provides a model for cone-specific degeneration; useful for drug and gene therapy screening.	[15]
cep290 mutation	Leber Congenital Amaurosis (LCA)	Recapitulates ciliopathy-related retinal degeneration; enables study of early-onset blindness.	[16]
aipl1b knockout	Leber Congenital Amaurosis (LCA)	Used to investigate protein stability in phototransduction and validate gene replacement therapies.	[16]
cngb3 mutation	Cone–Rod Dystrophy/Achromatopsia	Mimics cone dysfunction and degeneration; enables exploration of cone survival mechanisms.	[19]
mfrp mutation	Nanophthalmos/Retinal Degeneration	Provides insights into ocular size regulation and secondary retinal pathology.	[76]
myocilin (myoc) mutation	Glaucoma	Used to study intraocular pressure dysregulation and optic nerve degeneration.	[9]
usherin, harmonin mutations	Usher Syndrome	Helps dissect the role of ciliary and synaptic dysfunction in combined hearing and vision loss.	[19]

## Data Availability

No new data was created for this work.
